# Comparative Transcriptomic Analysis of Primary Duck Hepatocytes Provides Insight into Differential Susceptibility to DHBV Infection

**DOI:** 10.1371/journal.pone.0149702

**Published:** 2016-02-22

**Authors:** Liang Yan, Su Qu, Gang Liu, Lei Liu, Yao Yu, Guohui Ding, Yanfeng Zhao, Yixue Li, Youhua Xie, Junqi Zhang, Di Qu

**Affiliations:** 1 Key Laboratory of Medical Molecular Virology, Ministry of Education and Health, School of Basic Medical Sciences, Fudan University, Shanghai, China; 2 Department of Biochemistry and Molecular Biology, School of Basic Medical Sciences, Fudan University, Shanghai, China; 3 Key Laboratory of Systems Biology, Shanghai Institutes for Biological Sciences, Chinese Academy of Sciences, Shanghai, China; 4 Department of Laboratory Medicine, the Second Affiliated Hospital of Nanjing Medical University, Nanjing, Jiangsu, China; Indiana University, UNITED STATES

## Abstract

Primary duck hepatocytes (PDH) displays differential susceptibility to duck hepatitis B virus when maintained in the media supplemented with fetal bovine serum or dimethyl sulfoxide (DMSO) which has been widely used for the maintenance of hepatocytes, and prolonging susceptibility to hepadnavirus. However the mechanism underlying maintenance of susceptibility to hepadnavirus by DMSO treatment remains unclear. In this study, a global transcriptome analysis of PDHs under different culture conditions was conducted for investigating the effects of DMSO on maintenance of susceptibility of PDH to DHBV *in vitro*. The 384 differential expressed genes (DEGs) were identified by comparisons between each library pair (PDHs cultured with or without DMSO or fresh isolated PDH). We analyzed canonical pathways in which the DEGs were enriched in Hepatic Fibrosis / Hepatic Stellate Cell Activation, Bile Acid Biosynthesis and Tight Junction signaling. After re-annotation against human genome data, the 384 DEGs were pooled together with proteins belonging to hepatitis B pathway to construct a protein-protein interaction network. The combination of decreased expression of liver-specific genes (CYP3A4, CYP1E1, CFI, RELN and GSTA1 et al) with increased expression of hepatocyte-dedifferentiation-associated genes (PLA2G4A and PLCG1) suggested that *in vitro* culture conditions results in the fading of hepatocyte phenotype in PDHs. The expression of seven DEGs associated with tight junction formation (JAM3, PPP2R2B, PRKAR1B, PPP2R2C, MAGI2, ACTA2 and ACTG2) was up-regulated after short-term culture *in vitro*, which was attenuated in the presence of DMSO. Those results could shed light on DHBV infection associated molecular events affected by DMSO.

## Introduction

Hepatitis induced by hepatitis B virus (HBV) infection remains a major public health problem with over 350 million chronically infected carriers worldwide [1]. The lack of an appropriate cell model supporting HBV infection has been a major limitation for the study of the pathogenesis of hepadnavirus infection. NTCP has been demonstrated to be the functional receptor of HBV on the cell membrane recently [2]. However, more detailed interactions between hepadnavirus and hepatocytes need to be further investigated. Because of their susceptibility to DHBV infection, primary duck hepatocytes (PDH) serve as a valuable cell model to elucidate the key features of hepadnaviral infection [3–7]. A reverse transcribed RNA intermediate in the replication of hepadnavirus was first described in the DHBV-PDH infection model [3]. However, cultured *in vitro* over a period of time, PDH lose the hepatocyte phenotype and their susceptibility to DHBV infection rapidly diminishes [4,8]. Only 10%-20% cells retain susceptibility to DHBV infection 6 d after plating under *in vitro* culture condition. The rapid loss of susceptibility to DHBV infection has limited the application of PDH in hepadnavirus study [8].

Several modified culture methods have previously been developed for the maintenance of the primary hepatocyte, including the addition of a low concentration of DMSO to the culture medium [9,10], using plates pre-coated with rat-tail collagen [11], or supplementation with cell factors (epidermal growth factor or hepatocyte growth factor) [9]. Among these methods, the addition of DMSO has been widely used for the maintenance of hepatocytes, and prolonging susceptibility to hepadnavirus [12]. It shows differential susceptibility to DHBV infection between PDH maintained in culture media supplemented with 5% fetal bovine serum and 1.5% dimethyl sulfoxide (DMSO) [8]. It has been revealed that maintenance of polarization phenotypes and prevention of tight junction formation in primary human hepatocytes or HepaRG cells by DMSO is essential for the entry of the HBV under *in vitro* culture conditions [13]. However the mechanism underlying maintenance of susceptibility to hepadnavirus by DMSO remains to be elucidated.

Next-generation sequencing technology provides a useful tool to understand the activation patterns of the cellular response to external stimuli. Here we conducted a global transcriptome analysis of PDH under different culture conditions to investigate the effects of DMSO on maintenance of susceptibility of PDH to DHBV *in vitro*. The differential expressed genes that were identified by comparison between PDHs cultured with or without DMSO, were pooled together with proteins belonging to hepatitis B pathway for the construction of a protein-protein interaction (PPI) network. In the network, the expression of a cluster DEGs associated with tight junction formation was up-regulated after short-term culture *in vitro*, while the up-regulation was attenuated in the presence of DMSO. Supplementation with DMSO also relieved the declining of anti-virus associated DEGs expression in PDHs *in vitro*. Those results could shed light on DHBV infection associated molecular events affected by DMSO.

## Results

### Differential susceptibility to DHBV infection of primary duck hepatocytes maintained under different conditions

**Fig 1:**
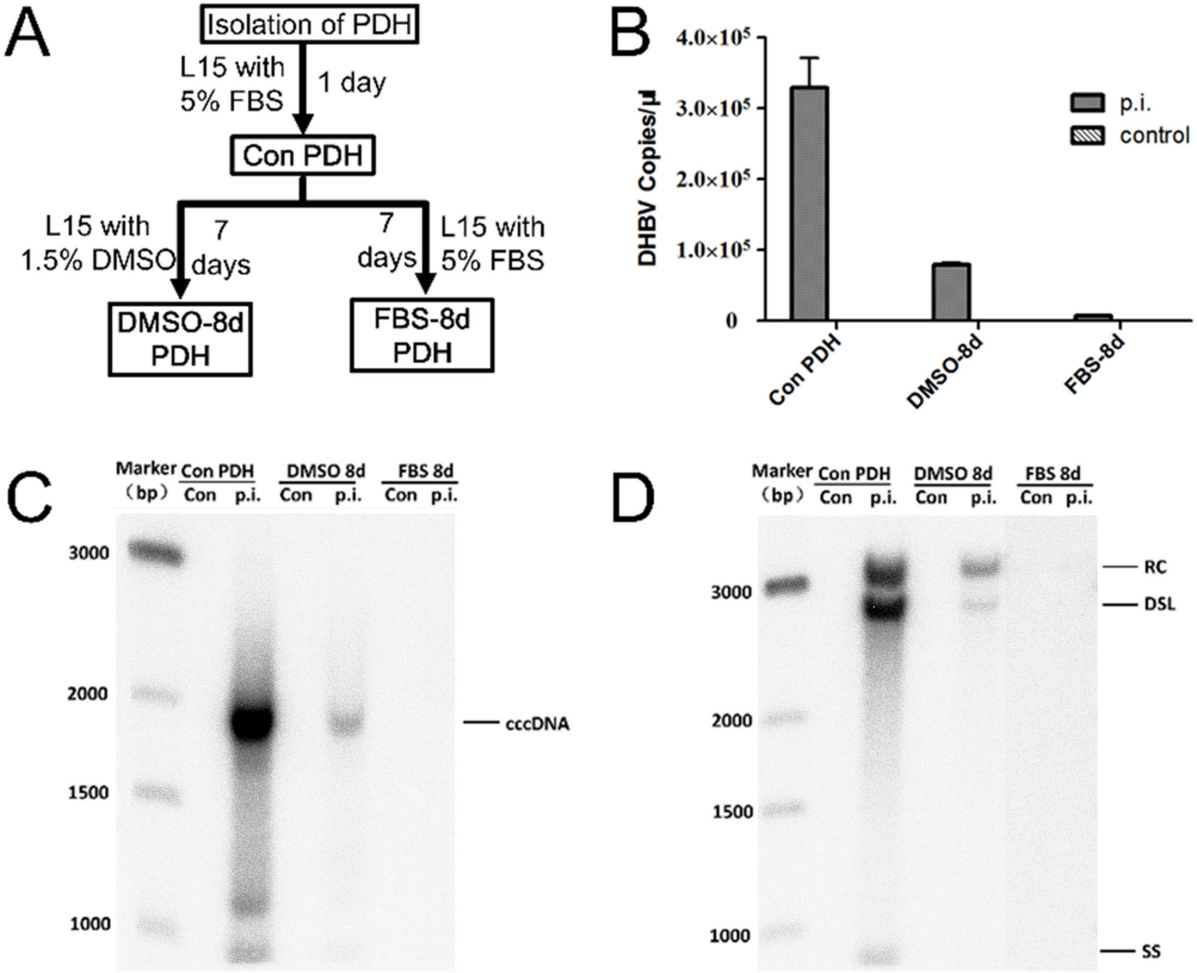
Intracellular and extracellular viral DNA detection for PDH maintained in different culture media. (A) The Con-PDH, DMSO-8d PDH and FBS-8d PDH (Two lanes of FBS 8d were transformed from a different gel.) were infected with DHBV at a multiplicities of genome equivalents (MGE) of 100 on the 1st or 8th d post-inoculation. (B) The supernatants from the PDH maintained in the different culture media were collected and DHBV DNA was extracted and quantified by qPCR. The cccDNA (C) and core DNA (D) were extracted and analyzed by Southern blot hybridization. RC, relaxed circular DNA; DSL, double-stranded linear DNA; SS, single-stranded DNA; cccDNA, covalently closed circular DNA.

In order to confirm susceptibility to DHBV infection, PDH isolated from Cherry Valley ducklings were maintained in Leibovitz's L-15 Medium supplemented with 5% FBS for 1d (Con-PDH), and then followed by culture in media with 5% FBS (FBS-8d PDH) or 1.5% DMSO (DMSO-8d PDH) for another 7 days (Fig 1A). The PDH under different culture conditions were infected with DHBV at a multiplicity of genome equivalents of 100, and maintained in media with 5% FBS for another 5 days. DHBV in the supernatant of Con-PDH was 3.29×10^5^ copies/μl, 7.89×10^4^ copies/μl in that of the DMSO-8d PDH, and only 6.72×10^3^ copies/μl in that of FBS-8d PDH, determined by qPCR (Fig 1B). DHBV covalently closed circular DNA (cccDNA) and DHBV DNA in the core particles (core DNA) were extracted from the cells and analyzed by Southern blot assay. The highest level of cccDNA and core DNA were detected in Con-PDH, followed by DMSO-8d PDH. The lowest level was detected in FBS-8d PDH (Fig 1C and 1D). It confirmed that treatment with 1.5% DMSO could maintain the susceptibility of the PDH to DHBV infection [8].

### Transcriptome analysis of primary duck hepatocytes under three culture conditions

Transcriptomes of PDH with differential susceptibility to DHBV infection were analyzed using RNA-seq. The sequencing libraries were prepared in triplicates for PDH under three different culture conditions (Con-PDH, DMSO-8d PDH and FBS-8d PDH), and a total of 220.25 million raw reads were generated. After the removal of ambiguous and low-quality reads (Q20<20), 195.74 high-quality reads were obtained and 173.89 million were further mapped onto the *Anas platyrhynchos* genome *(GenBank accession*: *PRJNA46621)* (Table 1).

**Table 1:** Summary of sequencing reads mapped to the *Anas platyrhynchos* reference genome.

Sample Name	PDH from duckling (NO.)	Raw Data		Processed Data		Mapping data	
		Total Reads (million)	Total Bases (Gb)	Total Reads (million)	Total Bases (Gb)	Mapped Reads (million)	Mapping Rate
	1	26.40	2.22	23.26	2.04	20.79	0.89
**Con-PDH**	2	18.92	1.58	16.59	1.45	14.63	0.88
	3	20.63	1.72	18.06	1.58	16.01	0.89
**DMSO-8d**	1	28.49	2.39	25.07	2.20	22.48	0.90
**PDH**	2	20.53	1.72	18.06	1.58	16.21	0.90
	3	32.76	2.83	30.08	2.68	26.48	0.88
**FBS-8d**	1	23.63	1.98	20.72	1.81	18.48	0.89
**PDH**	2	25.78	2.16	22.63	1.98	20.03	0.89
	3	23.10	2.00	21.26	1.90	18.78	0.88
**Total**		220.25	18.61	195.74	17.22	173.89	0.89

**Fig 2:**
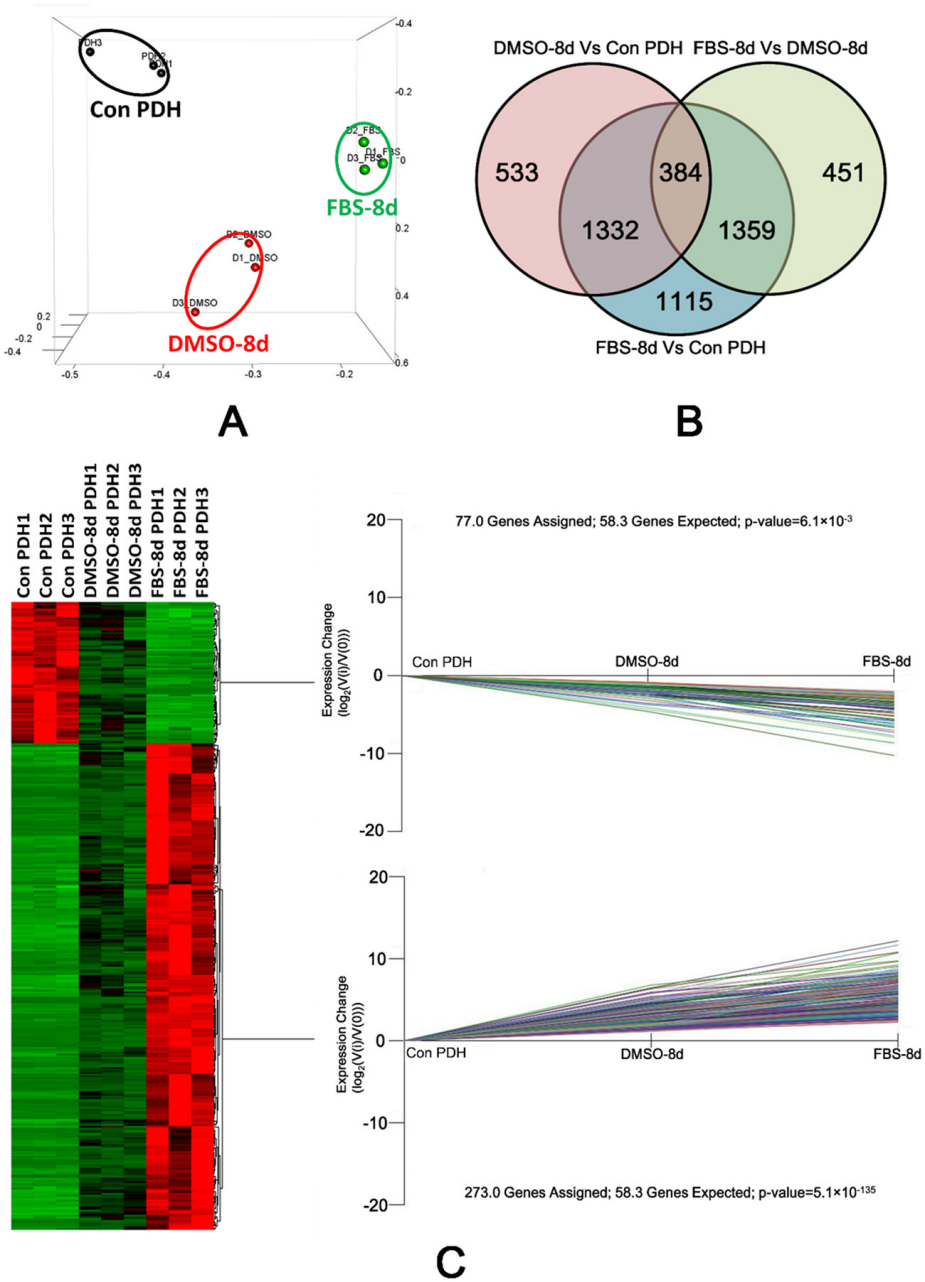
Expression pattern analysis of DEGs by PCA plot, Venn diagram and K-mean clustering. (A) PCA analysis of the transcriptomic data from RNA-seq. Single biological replicates are represented by dots, and PDH under different culture conditions are designated using separate colors. (B) Venn diagram of DEGs under different culture conditions. Three comparisons were performed between each library pair (Con-PDH, FBS-8d and DMSO-8d) and genes with an adjusted P value less than 0.05 and a change in transcription level of at least 2-fold were deemed to be differentially expressed. Among the comparisons, the number of overlapping genes is shown by the Venn diagram. (C) A heat map of the DEGs in PDH under various culture conditions. The columns represent triplicate samples of PDH under the three culture conditions and the relative expression level of genes are indicated by the color. Two significant K-mean clustering of the genes according to their expression pattern are shown.

Principal component analysis (PCA) was applied to analyze the sample data from PDHs under the different culture conditions (3 samples for each condition). It showed that transcriptome profiles of the samples from the same culture condition were clustered together (Fig 2A). We further compared the differentially expressed gene of PDH under three different culture condition (Con-PDH, DMSO-8d and FBS-8d). A Gene with an FDR (False discovery rate) adjusted P value less than 0.05 (t-tests) and at least 2-fold change in transcript level were deemed to be differentially expressed. By three comparisons between each library pair (FBS-8d vs Con-PDH, DMSO-8d vs Con-PDH and FBS-8d vs DMSO-8d PDH), 4190, 2249 and 2194 genes were identified as DEGs, respectively. A total of 384 DEGs were overlapped, as illustrated by a Venn-diagram (Fig 2B). Of those overlapping DEGs, expression of 298 genes were up-regulated and that of 86 genes down-regulated (S1 File). The overlapping DEGs were further analyzed using K-mean clustering. The 273 up-regulated and 77 down-regulated DEGs (were assigned to two significant expression patterns as Con-PDH < DMSO-8d < FBS-8d (P value: 5.1×10^−135^, Fisher’s exact test) and Con-PDH > DMSO-8d >FBS-8d (P value: 6.1×10^−3^, Fisher’s exact test), respectively (Fig 2C).

Expression of five genes in PDH under different culture conditions were selected for validation by quantitative reverse transcription PCR (RT-qPCR). Among them, two liver-specific expressed genes (*alb* encoding albumin and *ambp* for alpha-1-microglobulin/bikunin precursor) were identified as DEGs by RNA-seq, and three other genes (*cpd*, *gldc* and *furin* encoding carboxypeptidase D, glycine dehydrogenase and Furin, respectively) were not detected as DEGs but reported as proteins associated with DHBV infection [14–17]. Compared with that in FBS-8d PDH, PDH cultured under the culture condition supplemented with DMSO resulted in a 19-fold increase in expression of ALB and a 4-fold increase in AMBP determined by RNA-seq analysis (FPKM value), which was confirmed by RT-qPCR (Table 2). However, *in vitro* culture of PDH led to dramatic reduction of ALB and AMBP expression, whether or not DMSO was applied. For the DHBV-infection-associated-genes (*cpd*, *gldc* and *furin*), the presence of DMSO did not provide a distinct expression advantage over culture with FBS.

**Table 2:** Expression of five genes validated by RT-qPCR.

Genes	Functions of encoded proteins	Ratio detected by qRT-PCR*			Ratio detected by RNA-seq**		
		DMSO-8d/Con	FBS-8d/Con	DMSO-8d/FBS-8d	DMSO-8d/Con	FBS-8d/Con	DMSO-8d/FBS-8d
*alb*	Main protein of human blood plasma specifically synthesized in hepatocytes.	0.02	0.01	8.00	0.05	0.00	19.24
*ambp*	Belonging to the superfamily of lipocalin transport proteins and may play a role in the regulation of inflammatory processes.	0.09	0.02	4.50	0.50	0.12	4.30
*cpd*	DHBV docking receptor.	0.53	0.69	0.77	0.77	1.20	0.64
*gldc*	Involving in the membrane fusion of DHBV infection.	0.11	0.07	1.57	1.41	0.56	2.52
*furin*	An enzyme in the endosome which can cleave DHBV large surface protein.	0.95	0.46	2.07	0.53	0.72	0.73

* Relative expression of genes were calculated based on 2^-ΔΔCT^ using beta-actin as house-keeping gene.

**Ratio of FPKM value.

### Functional analysis and biological enrichment of DEGs

**Fig 3:**
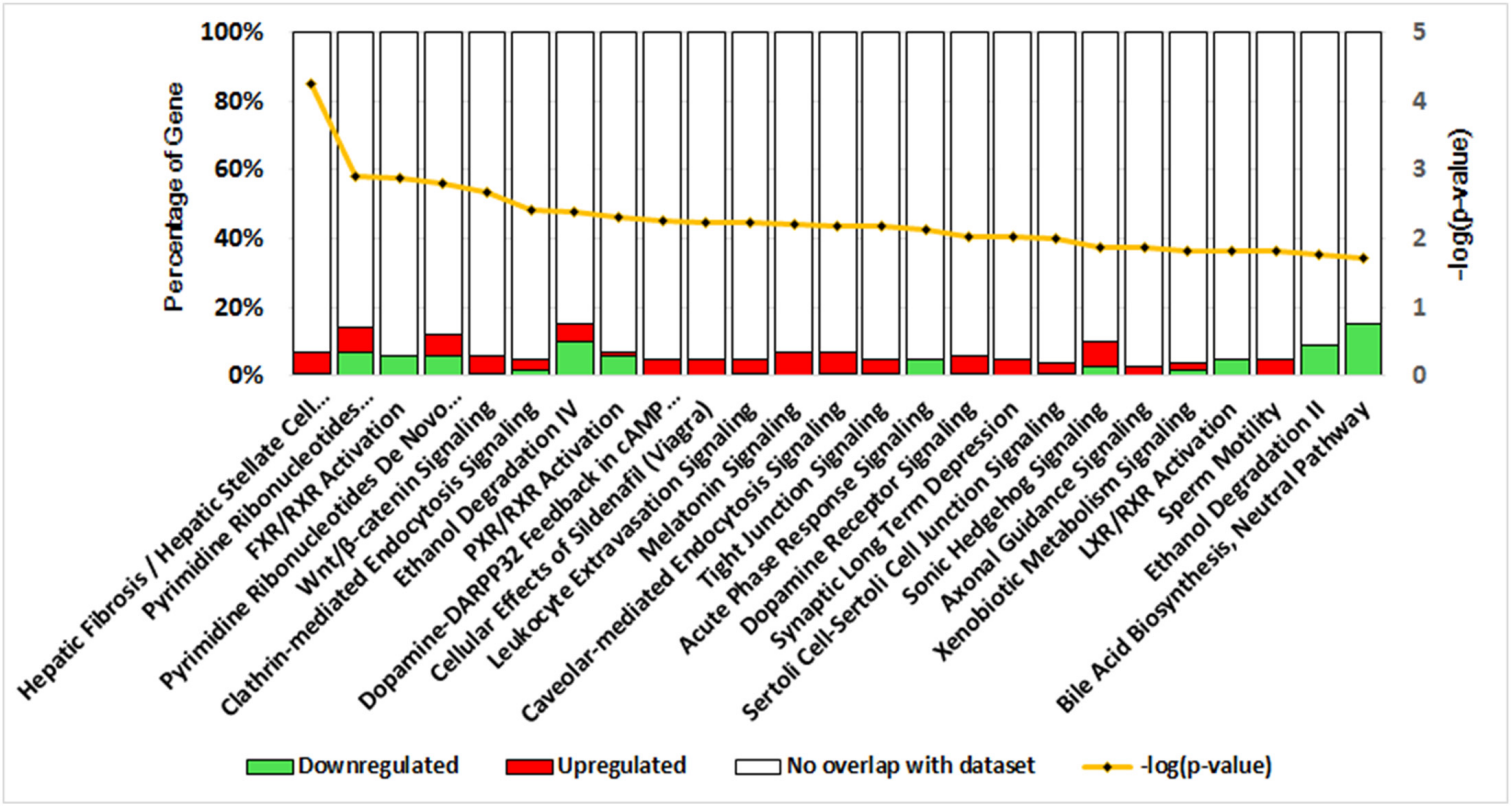
DEGs-associated canonical pathways as indicated by IPA. Canonical pathways were analyzed by IPA software using genes that were differentially expressed in PDH under the different culture conditions. The significance (P value, yellow line) of the pathways was calculated using Fisher’s Exact Test, and the percentages of down-regulated (green bar) and up-regulated genes (red bar), respectively, in each pathway are indicated.

To explore the biological behaviors of DEGs, DEGs were matched against the human genome using a BLAST search, with which 247 uncharacterized proteins in the *Anas platyrhynchos* genome *(GenBank accession*: *PRJNA46621)* were further annotated. Ingenuity Pathway Analysis (IPA) software was employed to analyze the deregulated canonical pathways. A total of 44 pathways were indicated (S2 File) and the 25 most significant pathways are shown in Fig 3. Among these pathways, Hepatic Fibrosis / Hepatic Stellate Cell Activation (P value: 5.75×10^−5^, Fisher’s exact test) was the most significant overrepresented pathway, followed by Pyrimidine Ribonucleotides Interconversion (P value: 1.25×10^−3^, Fisher’s exact test) and FXR/RXR Activation (P value: 1.28×10^−3^, Fisher’s exact test). The ratios of DEGs in each canonical pathway ranged between 3.2% and 15.4%. Most importantly, up-regulated or down-regulated genes accounted for the majority of the DEGs in pathways including Hepatic Fibrosis / Hepatic Stellate Cell Activation (up/down: 10/2, P value: 5.75×10^−5^), Ethanol Degradation II (up/down: 0/3, P value: 1.70×10^−2^) and Bile Acid Biosynthesis (up/down: 0/2, P value: 1.90×10^−2^). In addition, DEGs in Tight junction signaling (up/down: 7/1, P value: 6.76×10^−3^), Caveolar-mediated Endocytosis Signaling (up/down: 4/1, P value: 6.46×10^−3^) and Clathrin-mediated Endocytosis Signaling (up/down: 6/3, P value: 3.80×10^−3^) indicated the potential association with PDH susceptibility to DHBV.

### Key DEGs in DHBV infection revealed by a protein-protein interaction network

**Fig 4:**
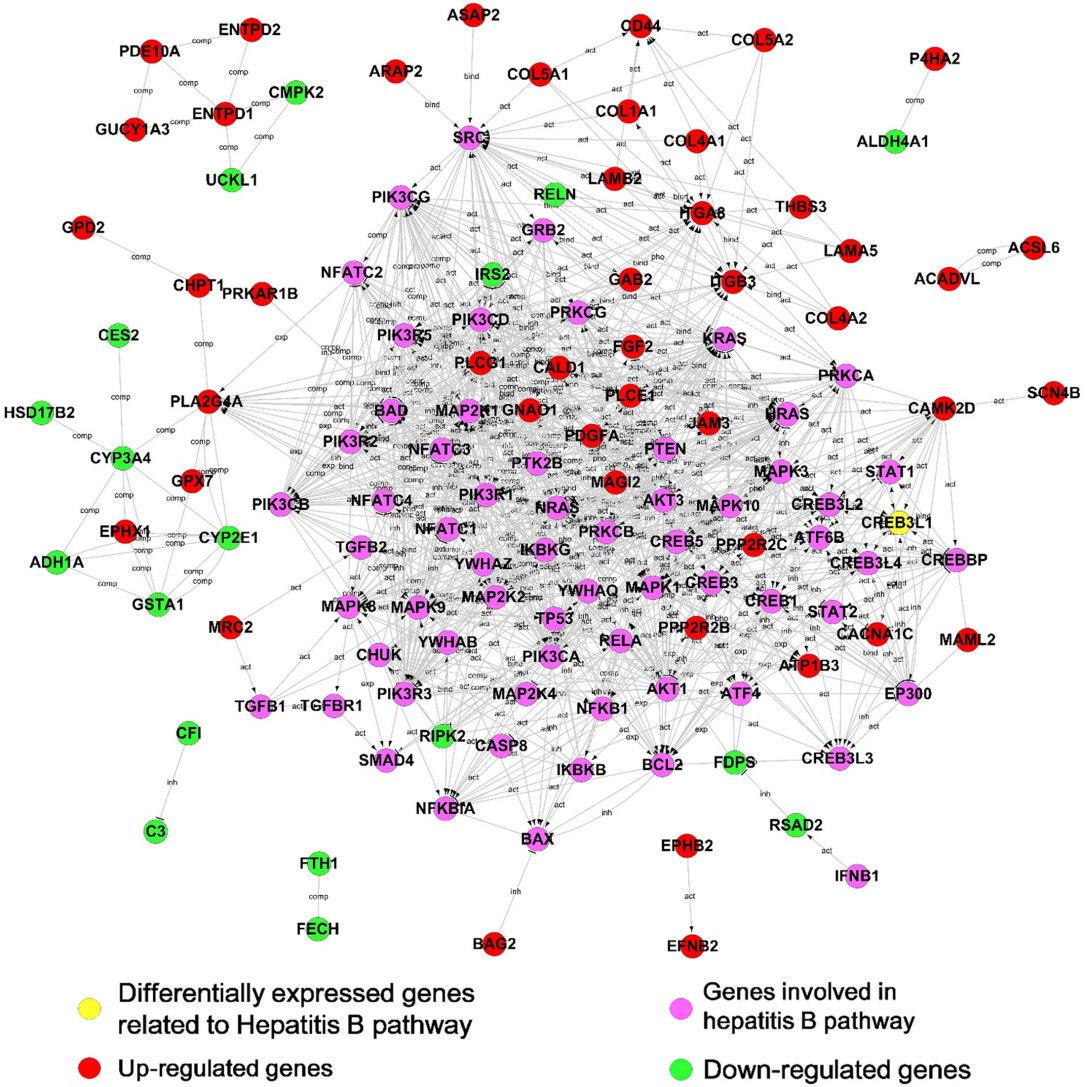
Interaction networks between DEGs and genes involved in the Hepatitis B pathway. The proteins encoded by DEGs (red, green or yellow nodes) or involved in the Hepatitis B pathway (purple nodes) in the KEGG database were extracted to construct a protein–protein interaction network. The lines in the network represent protein–protein interactions, including binding/association, phosphorylation, activation, and inhibition. Proteins encoded by up-regulated or down-regulated DEGs are indicated in red or green, respectively.

Hepadnavirus infection in hepatocytes is a complex, multi-step process mediated by diverse proteins. To determine links between the DEGs and hepadnavirus infection, a protein-protein interaction (PPI) network was constructed based on the KEGG database. Due to the limited interaction information for duck proteins, a human protein interaction database was applied to construct the network. The proteins encoded by DEGs were pooled with 113 proteins in hepatitis B (http://www.genome.jp/dbget-bin/get_linkdb?-t+orthology+path:map05161) as candidates of PPI network. The protein-protein interaction network illustrates 65 out of 384 proteins encoded by the DEGs and 48 out of 113 proteins encoded by genes belonging to hepatitis B pathway. In the network, 49 proteins encoded by DEGs showed a direct or indirect interaction with Hepatitis B pathway-associated proteins, and the others interact with each other among the DEGs. Most of the direct interactions were observed between proteins with similar alteration in expression under different culture conditions. Those up-regulated proteins interacted preferentially with proteins belonging to the Hepatitis B pathway. In the PPI network, 47 proteins encoded by DEGs were up-regulated (indicated as red nodes in Fig 4) and 18 ones were down-regulated (as green nodes).

The combination of decreased expression of liver-specific genes (CYP3A4, CYP1E1, CFI, RELN and GSTA1 et al, Table 3) with increased expression of hepatocyte-dedifferentiation-associated genes (PLA2G4A and PLCG1) indicates that *in vitro* culture conditions results in the fading of hepatocyte phenotype in PDHs. The presence of DMSO alleviated the hepatocyte phenotype fading and maintained the hepatocyte characters. The discrepancy of susceptibility of PDH to DHBV under different conditions was consistent with expression pattern of ferritin (FTH1) that was used as a predictor of host response to hepatitis B virus infection [18]. DEGs involving in virus infection were observed in the PPI network including CREB3L1, FDPS and IRS2 (Table 3), in which CREB3L1 was reported to be associated with iCell hepatocyte (induced pluripotent stem cell derived hepatocytes from Cellular Dynamics International) susceptibility to HBV infection [19]. It was unexpected that three genes (RSAD2, CMPK2, and RIPK2) playing roles in anti-viral immune responses (Table 3) showed down-regulated expression in PDHs under *in vitro* culture.

**Table 3:** Role of the differentially expressed genes in virus infection or cell process.

AccID	Gene Description (Duck)	Gene Description (Blast to Human)	FPKM value			Expression	Function indicated in virus infection or cell process	References
			**Con-PDH**	**DMSO-8d**	**FBS-8d**			
**Virus infection related DEGs**								
FTH1	Ferritin	Ferritin heavy chain	21370	8968	2102	down	Serum ferritin used as a predictor of host response to hepatitis B virus infection; HCV infection increased levels of serum ferritin.	Lustbader ED, et al., Science, 1983, 220:423–425 Garrido Serrano A, et al., Rev Esp Enferm Dig, 2001, 93:639–48 Shan Y, et al., Clin Infect Dis, 2005, 40:834–41
CREB3L1	Uncharacterized protein	highly similar to Homo sapiens cAMP responsive element binding protein 3-like 1 (CREB3L1)	1	2	25	up	Activated in response to virus infection to inhibit proliferation of virus-infected cells; proteolytically cleaved upon viral infection.	Denard B, et al., Cell Host Microbe, 2011, 10:65–74 Ye J,Biochim Biophys Acta, 2013, 1828:2926–32
FDPS	Uncharacterized protein	Farnesyl pyrophosphate synthase	405	189	73	down	Reduction of FPPS levels inhibited influenza virus release and replication.	Wang X, et al., Cell Host Microbe, 2007, 2:96–105.
IRS2	insulin receptor substrate 2	Insulin receptor substrate-2	8	3	1	down	Down-regulated by HCV infection.	Kawaguchi T, et al., Am J Pathol, 2004, 165:1499–508.
PLA2G4A	Uncharacterized protein	highly similar to Homo sapiens phospholipase A2, group IVA (cytosolic, calcium-dependent) (PLA2G4A)	0	5	53	up	Being crucial for the production of highly infectious HCV progeny; up-regulated in the cell lines infected with influenza virus.	Menzel N, et al., 2012, PLoS Pathog, 8: e1002829. Billharz R, et al., 2009, J Virol, 83:10557–10570.
**Liver-specific Genes**								
CFI	Uncharacterized protein	Complement factor I	22	7	1	down	An essential proteinase for regulating the complement cascade.	Ullman CG, et al., Mol Immunol, 1998, 35:503–12.
RELN	Uncharacterized protein	Reelin	6	3	0	down	Reduced expression of reelin (RELN) gene is associated with high recurrence rate of hepatocellular carcinoma.	Okamura Y, et al., Ann Surg Oncol, 2011, 18:572–9.
CES2	LOC101790670	Carboxylesterase 2 (intestine, liver)	64	14	1	down	Being responsible for the hydrolysis or transesterification of various xenobiotics and the major intestinal enzyme and functions in intestine drug clearance.	Taketani M, et al., Life Sci. 2007, 81(11):924–32
CYP3A4	LOC101799795	Cytochrome P450, family 3, subfamily A, polypeptide 4	552	22	0	down	Being the most abundantly expressed drug-metabolizing P450 enzyme in human liver and catalyze many reactions involved in drug metabolism.	Raucy JL, et al., Drug Metab Dispos. 2003, 31(5):533–9. Martínez-Jiménez CP, et al., Curr Drug Metab, 2007,8(2):185–94.
ADH1A	Uncharacterized protein	Alcohol dehydrogenase 1A (class I), alpha polypeptide	132	60	11	down	Catalyzing the oxidation of alcohols to aldehydes.	Lange LG, Biochemistry, 1976, 15(21): 4687–93.
GSTA1	LOC101797566	Glutathione S-transferase A1	601	209	9	down	Function in the detoxification of electrophilic compounds by conjugation with glutathione.	Karpusas M, et al., PLoS One. 2013, 8(2): e56337
CYP2E1	LOC101796596	Cytochrome P450 family 2 subfamily E polypeptide 1 (vase tunicate).	142	42	2	down	Catalyzing many reactions involved in drug metabolism and synthesis of cholesterol, steroids and other lipids.	Deka M, et al., World J Gastroenterol. 2010, 16(38):4800–8.
**Innate immune response related with anti-virus infection**								
RSAD2	Radical S-adenosyl methionine domain-containing protein 2	Highly similar to Homo sapiens viperin (cig5)	124	15	1	down	A cellular protein which could inhibit many DNA and RNA viruses such as CHIKV, HCMV, HCV, DENV, WNV, SINV, influenza, HIV LAI strain.	Teng TS, et al., J Clin Invest, 2012, 122:4447–60; Cho H, et al., Nat Med. 2013;19(4):458–64; Thomas E, et al., Gastroenterology, 2012,142:978–88
CMPK2	Uncharacterized protein	Mitochondrial cytidine monophosphate (UMP-CMP) kinase 2	75	22	2	down	Antiviral ISGs in primary neurons induced by IFN-βtreatment.	Li J, et al., Glia, 2011, 59:58–67
RIPK2	Uncharacterized protein	Highly similar to Receptor-interacting serine/threonine-protein kinase 2 (EC 2.7.11.1)	40	16	7	down	Modulated by HBeAg which supporting the concept that HBeAg could impair both innate and adaptive immune responses to promote chronic HBV infection; NOD2-RIPK2 signaling in protection against virally triggered immunopathology.	Wu S, et al., J Infect Dis.2012, 206:415–20; Lupfer C, et al., Nat Immunol, 2013, 14: 480–488
**Tight junction related DEGs**								
ACTA2	Uncharacterized protein	Highly similar to Actin, aortic smooth muscle	13	93	819	up	Playing a role in cell motility, structure and integrity.	Lee HW, et al., Clin Cancer Res, 2013, 19:5879–89.
JAM3	Uncharacterized protein	Junctional adhesion molecule C	0	2	9	up	Interact with the coxsackievirus and adenovirus receptor (CAR).	Mirza M, et al., Exp Cell Res. 2006, 312:817–30.
PPP2R2B	Serine/threonine-protein phosphatase 2A 55 kDa regulatory subunit B	Serine/threonine-protein phosphatase 2A 55 kDa regulatory subunit B beta isoform	0	2	22	up	Involved in the negative control of cell growth and division.	Cheng WT, et al., BMC Cell Biol, 2009,10:91
PRKAR1B	Uncharacterized protein	Protein kinase, cAMP-dependent, regulatory, type I, beta, isoform CRA_b	1	10	63	up	Controling many biochemical events in the cell including regulation of metabolism, ion transport, and gene transcription.	Elphinstone MS, et al., Clin Endocrinol (Oxf), 2004, 61:716–23.
PPP2R2C	Serine/threonine-protein phosphatase 2A 55 kDa regulatory subunit B	Serine/threonine-protein phosphatase 2A 55 kDa regulatory subunit B gamma isoform	0	3	10	up	Involved in the negative control of cell growth and division.	Backx L, et al., Eur J Med Genet, 2010, 53:239–43.
MAGI2	Uncharacterized protein	Highly similar to WW andPDZ domain-containing protein 2	0	0	1	up	Acting as scaffold molecule at synaptic junctions by assembling neurotransmitter receptors and cell adhesion proteins.	Wapenaar MC, et al., Gut, 2008, 57:463–7
ACTG2	Actin, gamma-enteric smooth muscle	cDNA FLJ42347 fis, clone UTERU2003399	13	101	271	up	Involved in various types of cell motility and in the maintenance of the cytoskeleton.	Citi S, et al., Ann N Y Acad Sci, 2009,1165:88–98
CLDN2	Claudin	Claudin-2	16	4	1	down	Expressed in an organ-specific manner and regulate tissue-specific physiologic properties of tight junctions.	Wu F, et al., Exp Eye Res, 2008, 87:214–25; Rosenthal R et al., J Cell Sci, 2010,123:1913–21; Van Itallie CM, et al., Mol Biol Cell, 2009, 20:3930–40.

Expression of seven DEGs associated with tight junction formation, including JAM3, PPP2R2B, PRKAR1B, PPP2R2C, MAGI2, ACTA2 and ACTG2, showed up-regulation pattern, which is in accordance with GO analysis of tight junction signaling pathway (P value: 1.25×10^−3^, Fisher’s exact test, Fig 3). Expression of those DEGs in DMSO-8d PDH was lower than that in FBS-8d PDHs, suggesting that DMSO-8d PDH had less tight junction formation that serves a blockade of HBV infection [13].

**Fig 5:**
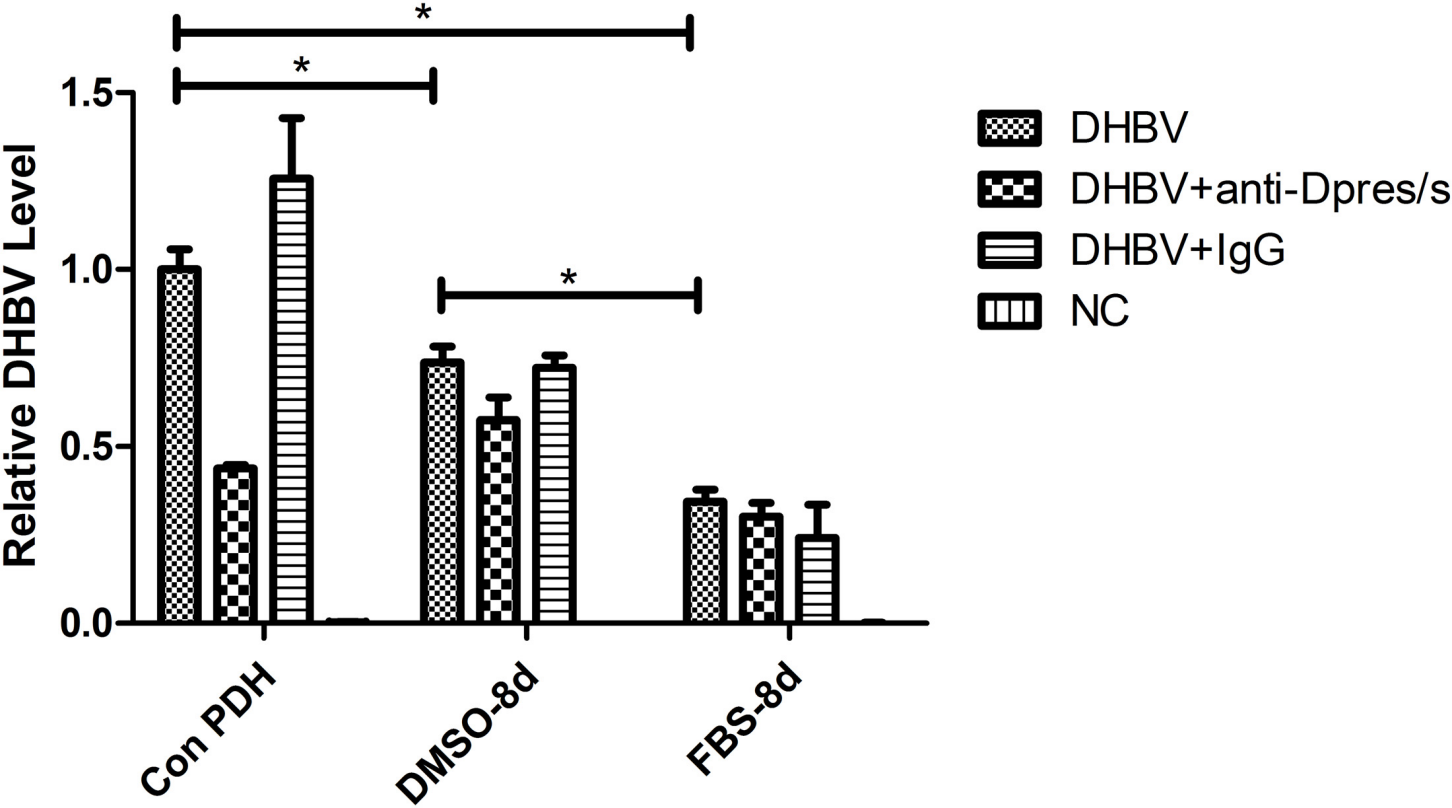
Differential DHBV attachment to PDH maintained under different conditions. PDH with differential susceptibility was pretreated with nocodazole and infected with DHBV at a MGE of 500. The DHBV attached to PDH were quantified by qPCR. Anti-Dpres/s or IgG were used as control. The asterisk represents a significant difference (t test, P value<0.05).

DHBV attachment to PDH with or without DMSO was carried out. DHBV were inoculated into PDH maintained under the three different culture conditions, and incubated at 4°C for 2 h. Anti-Pres/s of DHBV was used as control, which could block DHBV attaching to PDH. After washing of free virus, the attached DHBV were detected by qPCR. A decrease of DHBV attachment was observed in the presence of anti-Dpres/s for Con PDH. Attached DHBV in FBS-8d PDH (2.94-fold decrease) were, lower than that in DMSO-8d PDH (1.37-fold decrease), compared with Con PDH (Fig 5).

## Discussion

Hepadnaviruses exhibit a strong liver tropism and preferentially replicate in hepatocytes, the predominant liver cell type. Primary cultured hepatocytes remain the most important cell model used to elucidate the molecular pathogenesis underlying HBV infection. However, it remains unclear why primary hepatocytes gradually lose susceptibility to DHBV infection under *in vitro* culture conditions. Supplementation with DMSO can prolong PDH susceptibility to hepadnavirus [8]. In this study, DEGs were identified in PDH cultured under conditions with or without DMSO with RNA-seq analysis. Our results indicated that multiple factors were affected by DMSO treatment, and differential cell-cell junction may be one of the reasons leading to differential susceptibility in PDH *in vitro*.

For screening the genes of interest, we compared transcriptome data from PDHs cultured with 1.5% DMSO or 5% FBS, and fresh isolated PDH was used as a control. To identify related DEGs associated with PDH susceptibility to DHBV infection, a Venn diagram was used to visualize overlapping DEGs across the three comparisons (FBS-8d/Con-PDH, FBS-8d/DMSO-8d, DMSO-8d/Con-PDH). Among the DEGs (fold change>2 and FDR adjusted p-value<0.05) observed in the three comparisons, a total of 384 overlapping genes were obtained. Meanwhile, significant changes in 350 overlapping genes (273 up-regulated and 77 down-regulated genes) were enriched in two expression patterns (Con-PDH<DMSO-8d<FBS-8d and Con-PDH>DMSO-8d>FBS-8d), which provided further confirmation of a relationship between the DEGs and PDH susceptibility to DHBV infection. In another point of view, FPKM values of the down-regulated gene, which may be helpful in DHBV infection, are supposed to be higher than that of up-regulated ones. Therefore, FPKM values of the overlapped DEGs in Con PDH were compared by t-test. It indicated FPKM values of down-regulated gene were higher than that of up-regulated ones (p<0.0001, S1 Fig).

To obtain more protein information, DEGs were matched against the human genome using a BLAST search and 247 uncharacterized proteins were further annotated. Then Proteins encoded by overlapped DEGs and those from hepatitis B pathway were pooled together to construct a protein-protein interaction network. In the PPI network, a cluster of tight junction associated proteins (JAM3, PPP2R2B, PRKAR1B, PPP2R2C, MAGI2, ACTA2 and ACTG2) were up-regulated, suggesting that the formation of tight cell-cell junction between hepatocytes cultured *in vitro* over time. Since it has been assumed that tight junction formation creates a physical barrier preventing virus access to basolateral localized receptor(s), cell-cell junction alteration may contribute to differential susceptibility to DHBV between PDHs under different conditions. Under the culture condition with DMSO, tight junction formation between PDHs seemed to be retarded, evidenced by lower expression of tight junction genes than that in the absence of DMSO. The result will drive us to focus on molecular pathway of tight junction formation and virus entry in further investigation of influence of DMSO on hepadnavirus infection.

It is admitted that impacts of DMSO involve in more events of virus life cycle. Therefore, we analyzed the DHBV attachment, replication and release in PDH under different culture conditions. We have studied the effect of DMSO on DHBV attachment in PDHs (Fig 5) and virus replication in the LMH-D2 cells (S2 Fig). It showed that DHBV attachment and replication were increased in the presence of DMSO, indicating the extensive effect of DMSO on DHBV life cycle.

Differential susceptibility of PDH to DHBV also could be due to differential virus defense response to DHBV infection in PDHs under different culture condition. We observed down-regulated expression of anti-virus genes (RSAD2, CMPK2, and RIPK2), suggesting the collapse of cellular anti-virus immune concomitant with the loss of hepatic characters in PDH *in vitro*. It could rule out the possibility that weaken susceptibility of PDH to DHBV is the consequence of enhancement of cell defense against virus infection. In addition, expression of FTH1, a predictor of host response to hepatitis B virus infection *in vivo* [18] was down-regulated in FBS-8d PDH but partially recovered in DMSO-8d PDH. Expression of FTH1 could be used to predict availability of cell model for DHBV/HBV infection or to screen anti-virus infection chemicals.

We also analyzed the expression of genes which play important roles in the DHBV infection, such as *cpd*, *gldc* and *furin*. CPD is the docking receptor and binds to DHBV particles attached to heparin sulfate and co-factors on the hepatocyte membrane [14]. DHBV particles that bind to CPD are internalized into the cytoplasm, forming endosomes where the large protein on the DHBV membrane is cleaved by the Furin enzyme [15]. The cleaved large DHBV protein then binds to GLDC [16,17], followed by the membrane fusion of the DHBV particle and the endosome. NTCP is considered to be a functional receptor on the hepatocyte membrane and leads to the susceptibility of HepaG2 cell to HBV infection [2]. However, low FPKM value of NTCP was observed in PDH under different conditions (0.068, 0.36 and 0.28 in PDH-Con, DMSO-8d and FBS-8d, respectively) and *cpd*, *gldc*, *furin* and NTCP were not indicated as DEGs both by RNA-seq analysis and RT-qPCR.

Despite the candidate genes revealed in this study, further studies are still needed to investigate the association between these candidate genes and PDH susceptibility to DHBV infection. The mechanisms underlying the contribution of these genes to DHBV infection will be helpful for establishing new strategy against hepadnavirus infection.

## Conclusion

In this study, a global comparative transcriptomic analysis has been conducted to investigate differential susceptibility of PDH which were maintained under three conditions. A PPI network was constructed by pooling the DEGs with proteins belonging to hepatitis B pathway. The combination of decreased expression of liver-specific genes with increased expression of hepatocyte-dedifferentiation-associated genes suggested that the fading of hepatocyte phenotype of PDHs were resulted by *in vitro* culture conditions. At the same time, the expression of seven DEGs associated with tight junction formation was up-regulated, which was attenuated by DMSO. The study paves the way to fully understand mechanism underlying DHBV life cycle and to establish new strategy against hepadnavirus infection.

## Materials and Methods

### Ethics statement

All animal experiments with Cherry Valley ducklings that we performed in this study were approved by the Institutional Animal Care and Use Committee (IACUC) of Fudan University (NO.20150305).

### Experimental animals and cell lines

The 1-day-old Cherry Valley ducklings (*Anas domesticus*) were purchased from Breeding Center of Shanghai Institute of Veterinary Medical Sciences, Shanghai, China. The ducklings without of DHBV infection were used for PDH preparation, DHBV in the serum of ducklings were detected by qPCR with the specific primers (based on DHBV genome NC_001344.1), listed in Table 4.

**Table 4:** Primers for detection of DHBV DNA and differential expressed genes.

Gene	Sense	Primer Sequences	Primer Location	Designed based on
**DHBV**	F**	5'TACATTGCTGTTGTCGTGTG3'	2586–2605	DHBV genome
***pol***	R**	5'ATTGGCTAAGGCTCTAGAAG3'	2666–2685	NC_001344.1
	probe**	5'TGACTGTACCTTTGGTATGTACCATTG3'	2606–2632	
Alb*	F	5'TATCTGAGCATTGTGATTC3'	1485–1503	NM_00131039
	R	5'TGGTCTTCTGTTAGCATA3'	1572–1589	4.1
***ambp***	F	5'AATACACCTACTATAATCCTAA3'	308–329	XM_00501478
	R	5'CCATAACTACTTGTCTTCT3'	398–416	1.2
***cpd***	F	5'AAGCCATCATAGAGAACT3'	2582–2599	NM_00131038
	R	5'GGTAAGTGACAAGTAGAGA3'	2647–2665	2.1
***gldc***	F	5'AGTGCTATATTGCCTATT3'	2026–2043	NM_00131036
	R	5'GCTACATAACCTCTTACT3'	2166–2183	7.1
***furin***	F	5'GCAGAGGAAATGTGTCATC3'	1251–1269	XM_01310036
	R	5'CAGGCAGGCATCTACTTT3'	1315–1332	7.1
***actin***	F	5'TTCCAGCCATCTTTCTTG3'	784–801	NM_00131042
	R	5'GTCCACATCACACTTCAT3'	847–864	1.1

* Designed based on assembled transcripts in RNA-seq

**F: forward primer, R: reverse primer, Probe: FAM^™^ dye-labeled TaqMan MGB probe.

LMH-D2 cells (a generous gift of Dr. William Mason, Institute for Cancer Research, Philadelphia, Pa. USA), were maintained in DMEM F12 with 10% FBS and constitutively secreted DHBV virions into the supernatant [20].

### PDH preparation and culture

The PDH were isolated from the livers of 1-day-old cherry valley ducklings as previously described [21]. Briefly, DHBV-negative ducklings were anesthetized with 0.75% pelltobarbitalum natricum (10 mg/kg) and the portal vein was exposed. The liver was perfused with 80 ml of hepatocyte perfusion medium (Invitrogen, Carlsbad, CA, USA) and digested with collagenase IV (Sigma, St. Louis, MO, USA) via the portal vein. After removal of the blade, the liver was dispersed in hepatocyte wash medium (Invitrogen, Carlsbad, CA, USA), and the hepatocytes were separated from the non-parenchymal cells by centrifugation at 50×*g*. After washing, the hepatocytes were suspended in Leibovitz’s L-15 medium (Invitrogen, Carlsbad, CA, USA) supplemented with 5% FBS (Invitrogen, Carlsbad, CA, USA), 100 units/ml Penicillin-Streptomycin (Invitrogen, Carlsbad, CA, USA), 1 mg/L insulin (Sigma, St. Louis, MO, USA), and 10^−5^ M hydrocortisone-hemisuccinate (Sigma, St. Louis, MO, USA), and seeded at a density of 1×10^7^cells per 10 cm dish. The PDH were maintained at 37°C without CO_2_. After 24 h post-inoculation, the PDH were maintained in L-15 medium supplemented with 5% FBS or 1.5% DMSO for 7 d.

### Preparation and quantification of DHBV

For viral infection, virions of DHBV in the LMH D2 cell supernatants were precipitated with 10% PEG-8000 and dissolved in L-15 medium. The DHBV DNA in 200 μl L-15 medium was extracted with a TIANamp Virus DNA/RNA Kit (Tiangen, Beijing, China), followed by quantification using a TaqMan probe-based qPCR assay, which carried out with the primers and probe listed in Table 4. The qPCR amplification was performed with the Takara Probe PCR Kit on an Eppendorf RealPlex 4, using 50 cycles of a 2-step PCR program.

### DHBV infection and detection of viral cccDNA and core DNA

The PDH were maintained in Leibovitz's L-15 Medium supplemented with 5% FBS for 1 d (Con-PDH), and then followed by culture in media with 5% FBS (FBS-8d PDH) or 1.5% DMSO (DMSO-8d PDH) for 7 days. DHBV infection was carried out at a MGE of 100 by incubating overnight at 37°C. Then the inoculum was removed and L-15 medium supplemented with 5% FBS was added to the cells. On the 5^th^ day post-DHBV infection, PDH and culture supernatants were collected for isolation of DHBV cccDNA, and core DNA.

To isolate DHBV core DNA, PDH in a 35-mm-diameter dish were lysed with 0.5 ml of lysis buffer (1% NP-40, 0.15M NaCl, 50mM Tris-Cl 8.0, 1mM EDTA, 1×Protease inhibitor). Fifteen units of Micro Nuclease (Roche Diagnostics, Basel, Switzerland) were added to the lysate, incubated for 45 min at 37°C. The supernatants were collected by centrifuging at 14,000 rpm for 1 min, and the DHBV core particles were concentrated by adding PEG-8000 to a final concentration 10% (w/v). After centrifuging at 13,000 rpm for 5 min, the pellet was suspended in TNE lysis buffer (10 mM Tris-HCl, pH8.0, 100 mM NaCl, 1 mM EDTA, 0.5% SDS, 0.6 μg/μl proteinase K (Invitrogen, Carlsbad, CA, USA)), and incubated at 37°C for 1 h. The DHBV core DNA was extracted twice with Tris-buffered phenol/chloroform (pH 7.4), followed by one extraction with chloroform and precipitation by adding 3 volumes of 100% ethanol. The extracted DHBV core DNA was suspended in 20 μl of TE.

To isolate DHBV cccDNA, PDH were lysed in 1 ml of cccDNA extraction buffer containing 50 mM Tris-HCl, pH 8.0, 150 mM NaCl, 10 mM EDTA and 1% SDS. Then 250 μl of 2.5 M KCl was added to the lysate, rotated overnight at 4°C and centrifuged at 18,000 × *g* for 30 min at 4°C. The DHBV cccDNA was extracted from the supernatant 3 times with phenol and once with chloroform. After adding 2.2 volumes of absolute ethanol and incubating at -80°C overnight, precipitated DHBV cccDNA was centrifuged at 18,000 × *g* for 20 min, washed 3 times with 75% Ethanol and dissolved in 35 μl of TE buffer.

The extracted DHBV core or cccDNA was separated in a 1.2% agarose gel overnight at 30 v. The DNA transferred onto nitrocellulose membrane via upward capillary was detected by p32-labeled probes which were synthesized with a random-primed DNA Labeling Kit (Roche Diagnostics, Basel, Switzerland); the signals were exposed to a Phosphor screen for 24 h, and visualized by a PhosphorImager (Fujifilm, Kanagawa, Japan).

### Detection of DHBV attachment to PDH

The detection of DHBV attachment to PDH was described by Funk A et al [22]. In brief, PDH in 6-well plate were pretreated with 16.5 μM nocodazole for 1 h. The DHBV was inoculated at a MGE of 500 and incubated at 4°C for 2 h. DHBV premixed with anti-Dpres/s monoclonal antibody (1:1000) (a generous gift of Dr. William Mason in Fox Chase Cancer Center) or IgG (1:1000) were used as controls. After incubation with DHBV, the cells were washed with cold PBS for 5 times and lyzed with 200 μl lysis buffer (50 mM KCl, 10 mM Tris-HCl [pH 8.3], 0.45% Tween 20, 0.45% Nonidet P-40, 0.1 mg/ml proteinase K). The lysates were centrifuged at 16100 g for 10 min followed by DHBV DNA extraction with TIANamp Virus DNA/RNA Kit. Then DHBV DNA was quantified by qPCR.

### Preparation of the PDH cDNA library, and sequencing

For transcriptome analysis, cDNA libraries were established for the PDH samples. The total RNA was extracted from PDH under three culture conditions (in Leibovitz's L-15 Medium supplemented with 5% FBS for 1 d, followed by culturing in media with 5% FBS or 1.5% DMSO for 7 d) using Trizol reagent following the manufacturer’s protocol (Invitrogen, Carlsbad, CA, USA). Then, poly-A mRNA was isolated from 10μg total RNA using a TruSeq^®^ RNA Sample Preparation v2 kit (Illumina Inc., San Diego, CA, USA) and was fragmented at 94°C for 8 min. The first and second cDNA strands were synthesized successively, followed by the purification of double-stranded cDNA using AMPure XP beads (Beckman Coulter, Brea, CA, USA). After end repair, cDNA was adenylated at the 3′ end, ligated to the sequencing adapters and amplified by PCR. The quality and quantity of the cDNA library was determined with an Agilent DNA 1000 Kit and an Agilent 2100 Bioanalyzer (Agilent Technologies, Palo Alto, CA, USA). PDH cDNA libraries were sequenced on the Illumina Solexa platform using the pair-end approach and the Illumina data processing pipeline.

### Processing and mapping of Illumina reads

In order to rule out sequencing errors, all raw reads obtained from the Illumina platform were pre-processed prior to the next step. Clean reads were obtained by removing the adaptor sequences, reads with >5% ambiguous bases (noted as N) and low-quality reads containing more than 20 percent of bases with qualities of <20. High-quality reads were mapped to the *Anas platyrhynchos* genome and transcripts were downloaded from ENSEMBL (http://www.ensembl.org) with TopHat v2.0.12 software (http://ccb.jhu.edu/software/tophat/index.shtml). TopHat allows multiple alignments per read and the default parameters were used. Cufflinks v2.2.1 software (http://cufflinks.cbcb.umd.edu/) was later used for analyses that included transcript assembly and FPKM value calculations to quantify gene expression; this program was also run with default parameters. Genes with an FDR adjusted P value less than 0.05 and a change in levels of at least 2-fold were considered to be differentially expressed.

### Validation of DEGs by real-time PCR

For each RNA sample, 4 μg of total RNA was reverse-transcribed using the TIANScript II RT Kit (Tiangen, Beijing, China). The cDNA was diluted 1:10, and 2 μl of the cDNA was used as template in 20 μl of amplification reaction, using SYBR ^®^ Premix Ex Taq^™^ II according to the manufacturer’s protocols (Takara, Dalian, China) on a Mastercycler ep realplex (Eppendorf, Hamburg, Germany). The specific primers for each gene were designed using Beacon designer 7.5 software (Palo Alto, CA, USA), and are listed in Table 4.

### Canonical pathway analysis by IPA

The differentially expressed genes with corresponding expression values were uploaded to the IPA Software, and analyzed by canonical pathway. We used Fisher’s exact test to select the significant pathways and the significance threshold was defined by a P value of 0.05. The significance of the pathway was indicated by the ratio of the number of genes form the dataset that mapped to the pathway to the total number of genes present in the canonical pathway.

### Construction of protein-protein interaction network

A protein-protein interaction network was constructed using Cytoscape software. The candidate proteins are encoded by DEGs in the present study and from proteins involved in hepatitis B pathway in KEGG (http://www.genome.jp/dbget-bin/get_linkdb?-t+orthology+path:map05161). A total number of 113 genes which were reported involved in hepatitis B pathway were extracted. Due to the limited interaction information for duck proteins, DEGs identified by RNA-seq analysis were matched against the human genome using BLAST method, and matched ones were pooled together as candidate genes. The protein–protein interaction network was constructed according to the functional relationships annotated in the KEGG database. In the network, lines were used to represent protein–protein interactions including binding/association, phosphorylation, activation, and inhibition. Proteins encoded by up-regulated or down-regulated DEGs were indicated in red or green, respectively, while purple nodes were used to present proteins involved in the hepatitis B pathway.

### Statistical analysis

T test was used to filter the differentially expressed genes, after the significant analysis and FDR analysis under the following criteria: i) Fold Change>2 or <0.5; ii) FDR<0.05. Fisher’s exact test was used in K-means clustering and Canonical pathway analysis. A P value<0.05 was considered to be statistically significant.

## Supporting Information

S1 Fig Comparison of FPKM values of up- and down-regulated DEGs in Con PDH. The FPKM values overlapped up- and down-regulated genes in Con PDH were compared by t-test.
(TIF)

S2 Fig Effects of DMSO on DHBV replication in LMH D2 cell. The LMH D2 cell were cultured in DMEM-F12 medium with 0.5% or 1.5% DMSO. LMH D2 cell under normal culture conditions were used as control. The cccDNA and core DNA were extracted on the 1st, 3rd, 5th and 7th day post DMSO treatment and detected by Southern Blot. D: DMSO group; C: Control group.
(TIF)

S1 File Differential expressed genes associated with PDH susceptibility to DHBV infection. (XLSX)

S2 File Canonical pathways analysis of differential expressed genes by IPA Software. (XLS)
