# Supplementary figures and images for: Comparative Transcriptomic Analysis of Primary Duck Hepatocytes Provides Insight into Differential Susceptibility to DHBV Infection
Source: PLoS One. 2016 Feb 22;11(2):e0149702. doi: 10.1371/journal.pone.0149702 (PMC4763071; doi:10.1371/journal.pone.0149702)

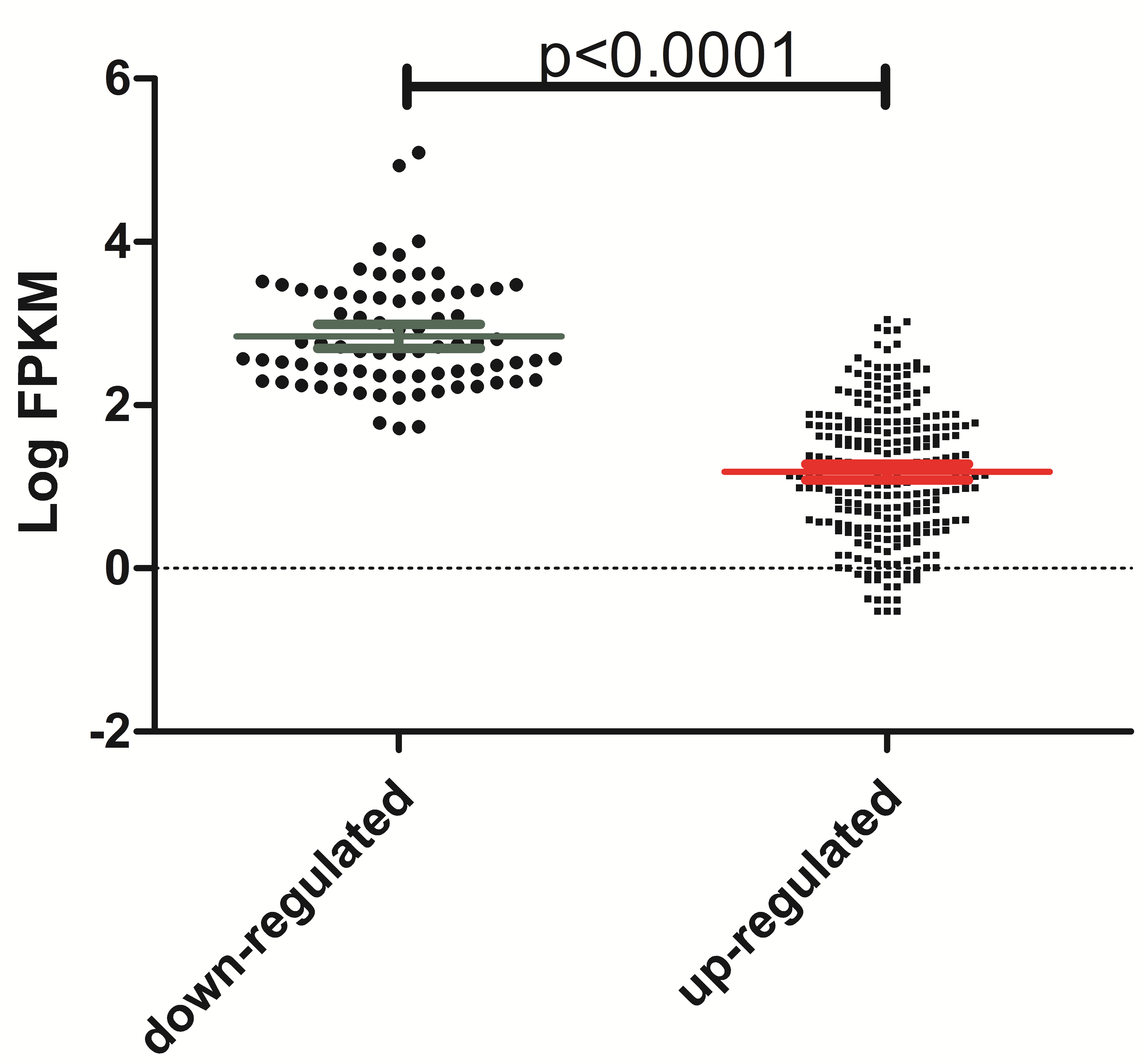

Supplement: S1 Fig — The FPKM values overlapped up- and down-regulated genes in Con PDH were compared by t-test. (TIF) [file pone.0149702.s001.tif]

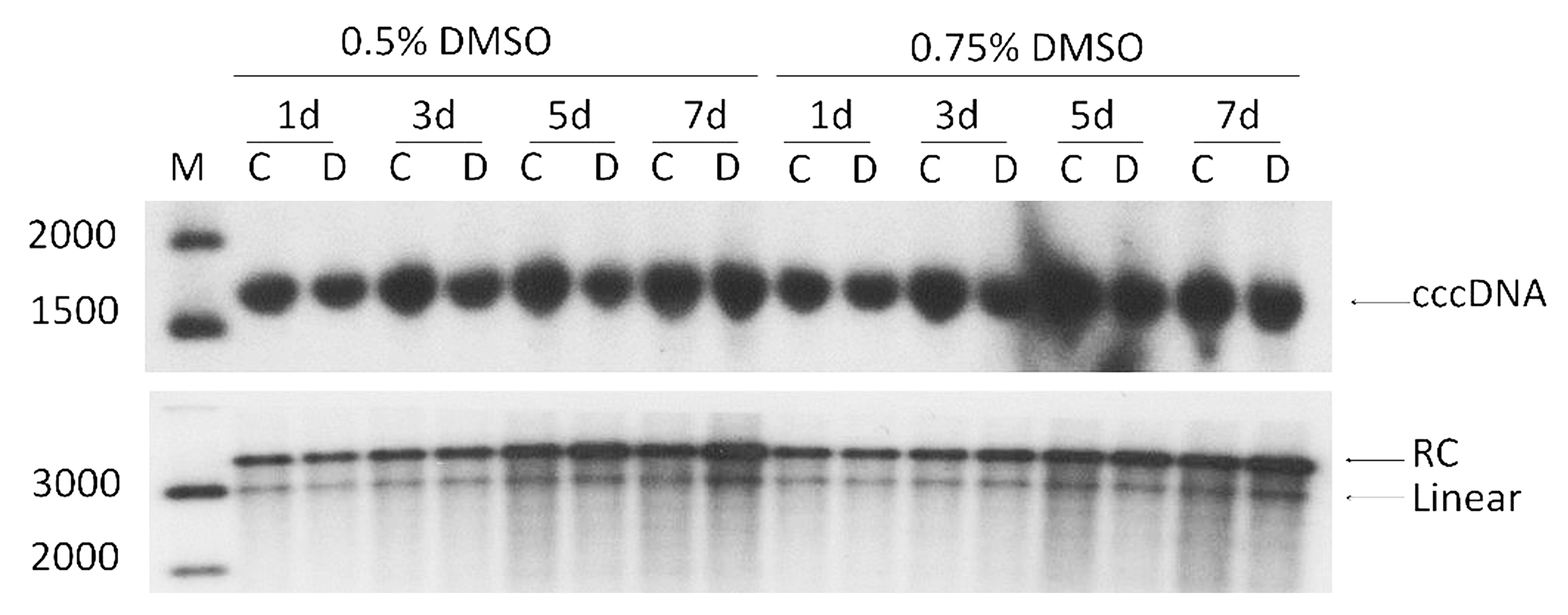

Supplement: S2 Fig — The LMH D2 cell were cultured in DMEM-F12 medium with 0.5% or 1.5% DMSO. LMH D2 cell under normal culture conditions were used as control. The cccDNA and core DNA were extracted on the 1st, 3rd, 5th and 7th day post DMSO treatment and detected by Southern Blot. D: DMSO group; C: Control group. (TIF) [file pone.0149702.s002.tif]
